# Jitteriness/Anxiety Syndrome Developing Immediately following Initiation of Oral Administration of Sertraline

**DOI:** 10.1155/2017/1319505

**Published:** 2017-08-22

**Authors:** Toshinori Nakamura, Nobuhiro Sugiyama, Daimei Sasayama, Tetsuya Hagiwara, Shinsuke Washizuka

**Affiliations:** ^1^Department of Psychiatry, Shinshu University School of Medicine, 3-1-1 Asahi, Matsumoto, Nagano 390-8621, Japan; ^2^Department of Psychiatry, Shinshu University School of Health Sciences, 3-1-1 Asahi, Matsumoto, Nagano 390-8621, Japan

## Abstract

Here, we report our experience with patients in whom jitteriness/anxiety syndrome developed immediately following the start of oral sertraline administration. Administration was discontinued in these patients on day 2, and the jitteriness/anxiety syndrome improved the following day. Jitteriness/anxiety syndrome may develop immediately following oral administration of even low doses of sertraline, and improvement can be expected if sertraline is promptly discontinued.

## 1. Introduction

According to the United States Food and Drug Administration (FDA) guidelines, jitteriness/anxiety syndrome was referred to as “activation syndrome.” The following ten symptoms of jitteriness/anxiety syndrome were listed: anxiety, agitation, panic attacks, insomnia, irritability, hostility, aggressiveness, impulsivity, akathisia (severe restlessness), and (hypo)mania. These symptoms were considered to be associated with an increased risk for suicide attempts [1]. Although jitteriness/anxiety syndrome is known to be potentially caused by the use of antidepressants in general, it occurs rarely in patients specifically treated with sertraline. In a prior study, Pohl et al. reported no significant differences in the occurrence between patients administered sertraline and those administered placebo [2]. However, we encountered two cases in which both the patients developed jitteriness/anxiety syndrome on the day after initiation of a regimen of sertraline. To our knowledge, this is the first report on the onset of jitteriness/anxiety syndrome caused by a low dose of sertraline.

## 2. Cases

### 2.1. Case Presentation 1

A man in his 1950s without a history of (hypo)manic episodes, drug/alcohol abuse, and anamnestic characteristics or a family history of mental illness presented with loss of motivation, disordered sleep, a depressed mood, decreased appetite and libido, a general loss of interest, headache, palpitation, and chills, which had persisted for 3 months. We diagnosed depression and initiated an oral administration of sertraline 25 mg after supper. However, the patient exhibited anxiety, irritation, and akathisia beginning in the morning of the next day. After taking sertraline on day 2, the symptoms continued through the following day, and we subsequently discontinued administration on day three. A gradual remission of symptoms was noted in the days that followed.

### 2.2. Case Presentation 2

A woman in her 1940s without a history of (hypo)manic episodes, drug/alcohol abuse, and anamnestic characteristics or a family history of mental illness was brought to our hospital one night for emergency treatment of palpitations and dyspnea. She was suspected to have psychiatric issues, as she exhibited the same symptoms every night without cause. We diagnosed panic disorder and initiated oral administration of sertraline 25 mg after supper. Concomitant clotiazepam was to be used on an as-needed basis. Following administration of sertraline, she was unable to sleep and anxious. The following morning, she felt a sense of uneasiness and irritation, which she had never experienced before. Symptoms related to panic attacks, such as palpitations and dyspnea, were not apparent. She also presented with a dysarthria and malaise. The next day, she took sertraline 25 mg at 8:00 PM, following which she exhibited akathisia in addition to anxiety and irritation. She had strong malaise after arising the next morning and a marked loss of motivation. Sertraline administration was discontinued on day two. On the day of discontinuation, she slept well and her symptoms disappeared the following day.

## 3. Discussion

The man in his 1950s exhibited 3 of the 10 symptoms of jitteriness/anxiety syndrome—anxiety, irritation, and akathisia—while the woman in her 1940s exhibited 4 of them, including anxiety, irritation, insomnia, and akathisia. It can be concluded that jitteriness/anxiety syndrome and not an anxiety disorder was the cause of the female patient's anxiety because of the noted difference between the anxiety experienced during past panic attacks with palpitations and dyspnea, and the anxiety experienced during her use of sertraline.

Jitteriness/anxiety syndrome occurs with antidepressants in general [3], and there have also been reports of sertraline causing jitteriness/anxiety syndrome [4], though this is generally considered very rare [2]. However, we experienced two cases of jitteriness/anxiety syndrome developing immediately following the initiation of administration at low doses of sertraline.

Previous studies have shown that with the use of selective serotonin reuptake inhibitors, symptoms consistent with jitteriness/anxiety syndrome are exhibited within 12 hours of administration of an initial dose (20 mg) of fluoxetine [5] and 1 hour after the oral administration of escitalopram 5 mg [6]. Our female patient in her 1940s exhibited symptoms of jitteriness/anxiety syndrome in the evening of the initial day of oral administration, while the male patient in his 1950s had symptoms beginning in the morning after administration. The symptoms disappeared in both cases after the cessation of sertraline. The risk factors or vulnerability in these cases which led to the onset of jitteriness/anxiety syndrome remain to be investigated [3].

Although considered rare [2], the prevalence of sertraline-induced jitteriness/anxiety syndrome remains to be investigated. It might be overlooked in some cases, because such symptoms could be mistaken as psychiatric symptoms if psychiatrists are not aware of its occurrence. Thus, it is possible that careful medical interview may reveal higher prevalence than expected from previous reports. Correct recognition of antidepressant-induced jitteriness/anxiety syndrome is of great clinical importance, because the cessation of the administration of antidepressants will promptly ameliorate the symptoms. Psychiatrists should always bear in mind the possibility of the development of jitteriness/anxiety syndrome when prescribing sertraline. It should be noted that jitteriness/anxiety syndrome may develop rapidly after the initiation of oral administration of even low-dose sertraline.

## References

[1] Food and Drug Administration Worsening depression and suicidality in patients being treated with antidepressant.

[2] Pohl R. B., Wolkow R. M., Clary C. M. (1998). Sertraline in the treatment of panic disorder: a double-blind multicenter trial. *American Journal of Psychiatry*.

[3] Sinclair L. I., Christmas D. M., Hood S. D. (2009). Antidepressant-induced jitteriness/anxiety syndrome: systematic review. *British Journal of Psychiatry*.

[4] Guilé J.-M. (1996). Sertraline-induced behavioral activation during the treatment of an adolescent with major depression. *Journal of Child and Adolescent Psychopharmacology*.

[5] Lipinski J. F., Mallya G., Zimmerman P., Pope H. G. (1989). Fluoxetine-induced akathisia: clinical and theoretical implications. *Journal of Clinical Psychiatry*.

[6] Muhtz C., Agorastos A., Kellner M. (2009). Acute maniform reaction to a single dose of escitalopram in a social phobic patient—An atypical jitteriness syndrome?. *Pharmacopsychiatry*.

